# Dry Surface Biofilm Formation by *Candida auris* Facilitates Persistence and Tolerance to Sodium Hypochlorite

**DOI:** 10.1111/apm.70022

**Published:** 2025-04-07

**Authors:** Alicia Ware, William Johnston, Christopher Delaney, Mark C. Butcher, Gordon Ramage, Lesley Price, John Butcher, Ryan Kean

**Affiliations:** ^1^ Department of Biological and Biomedical Sciences Glasgow Caledonian University Glasgow UK; ^2^ Safeguarding Health Through Infection Prevention Research Group, Research Centre for Health (ReaCH) Glasgow Caledonian University Glasgow UK; ^3^ Oral Sciences Research Group University of Glasgow Glasgow UK

**Keywords:** biofilm, *Candida auris*, disinfection, infection prevention control, sodium hypochlorite

## Abstract

*Candida auris* is an enigmatic fungal pathogen, recently elevated as a critical priority group pathogen by the World Health Organisation, linked with its ability to cause outbreaks within nosocomial care units, facilitated through environmental persistence. We investigated the susceptibility of phenotypically distinct *C. auris* isolates to sodium hypochlorite (NaOCl), and evaluated the role of biofilms in surviving disinfection using a dry‐surface biofilm (DSB) model and transcriptomic profiling. Planktonic cells were tested for susceptibility to NaOCl, with biofilm formation using the 12‐day DSB model, assessed using viable counts, biomass assays and microscopy. Disinfection efficacy was assessed using clinical protocols of 500–1,000 ppm for 1–5 min. RNA sequencing was performed on untreated DSBs in comparison to planktonic cells. Isolates were found to be susceptible planktonically, but grew NaOCl‐tolerant biofilms, with only 2–4 log_10_ reductions in viable cells observed at highest concentrations. Transcriptomics identified DSB upregulation of ABC transporters and iron acquisition pathways relative to planktonic cells. Our findings optimized a DSB protocol in which *C. auris* can mediate tolerance to NaOCl disinfection, suggesting a lifestyle through which this problematic yeast can environmentally persist. Mechanistically, it has been shown for the first time that upregulation of small‐molecule and iron transport pathways are potential facilitators of environmental survival.

## 1 Introduction

In little over a decade, *Candida auris* has emerged as a significant nosocomial threat, responsible for outbreaks across the globe, and most recently exacerbated by the COVID‐19 pandemic [1]. In late 2022, the World Health Organisation highlighted *C. auris* as one of four fungal pathogens in the Critical Priority group, owing to its intrinsic resistance to certain antifungal agents and ability to cause life‐threatening infections resulting in unacceptably high mortality rates [1]. *C. auris* readily forms biofilms on biotic and abiotic surfaces in vitro [2, 3], a survival mechanism which is strongly suspected in persistence within the healthcare setting [4]. These communities of cells have routinely been shown to tolerate increased concentrations of all three classes of antifungals, facilitated through drug sequestration by the extracellular matrix (ECM) and upregulation of efflux pumps *CDR1* and *MDR1* [5, 6]. In vitro evidence also suggests that biofilms also facilitate skin colonisation, with *C. auris* displaying enhanced adhesive capacity on both human and porcine skin models in comparison to *C. albicans* [2]. Also, potentially linked to survival is the ability of *C. auris* isolates to exist as aggregative/non‐aggregative phenotypes that have been previously identified with differences in virulence and biofilm formation [7, 8]. Although environmental contamination is a likely reservoir for transmission of *C. auris* between hosts [9], the role biofilms may play in this process is less well studied.

One concept that links environmental contamination with hospital‐acquired infections is biofilm formation on dry surfaces [10]. Most extensively studied in the bacterial pathogen *Staphylococcus aureus* , these biofilms have been identified in situ on fomites within high‐contact areas such as patient folders, keyboards and sanitising bottles [10]. In vitro models have shown that these communities can withstand environmental stressors such as temperature [11], physical removal and disinfection [12]. Given the capacity for *C. auris* to survive on various substrates for extended periods [13], and published reports of clinical transmission facilitated by reusable equipment such as temperature probes [14], a function for biofilms facilitating environmental persistence and potential outbreaks is highly plausible.

Cleaning and disinfection of environmental contamination is therefore of key importance in preventing transmission and outbreaks of *C. auris*. While a number of studies have demonstrated concentration‐ and contact time‐dependent efficacy of biocides with various active agents when tested against *C. auris* in suspension [15, 16, 17, 18], biofilm testing has reported decreased efficacy of compounds such as sodium hypochlorite (NaOCl) [3]. Chlorine‐based oxidising agents such as NaOCl are widely regarded as the gold standard for disinfection of surfaces and equipment, owing to a wide spectrum of activity [19], with non‐contact‐based methods such as gaseous hydrogen peroxide, ozone or ultraviolet light providing supplemental disinfection strategies. These gaseous and non‐contact‐based methods have demonstrated good in vitro efficacy against *C. auris* in both planktonic and biofilm form in previous studies [20, 21, 22]. Given the knowledge gap in understanding survival strategies of *C. auris* in response to biocidal antimicrobial agents, we herein investigated the function and mechanism of biofilms mimicking those formed on nosocomial dry surfaces in response to sodium hypochlorite disinfection. Here we report for the first time that DSBs of *C. auris* clinical isolates from outbreak‐associated clades develop tolerance to NaOCl over subsequent cycles, potentially facilitated by upregulation of efflux pumps and iron scavenging mechanisms.

## 2 Materials and Methods

### 2.1 Microbial Culture and Standardisation

Clinical isolates of *C. auris* from two outbreak‐associated phylogenetic clades, which were not multi‐drug resistant, displayed varying biofilm‐forming capacity and included aggregating versus single‐celled phenotypes, were selected for this study (Table 1) [7]. Isolates were maintained on Sabouraud's dextrose agar (SAB [Sigma‐Aldrich]) following incubation for 48 h at 30°C. Overnight broths were prepared by inoculation of colonies into yeast peptone dextrose medium (YPD [Sigma‐Aldrich]) and incubation at 30°C for 18 h in an orbital shaking incubator at 150 rpm. Cells were quantified using a Neubauer haemocytometer and standardized to the required concentration in appropriate medium as detailed in sections below.

**TABLE 1:** Key characteristics of selected *Candida auris* strains.

Growth phenotype	Isolate	Biofilm former phenotype	Phylogeographic clade
Single‐celled	NCPF8990	High	I
	NCPF8973	Moderate	I
	Strain 174	Low	I
Aggregating	NCPF8991	High	I
	NCPF8979	Moderate	III
	Strain 182	Low	III

### 2.2 Dry Surface Biofilm Formation

A dry surface biofilm (DSB) protocol was adapted from Ledwoch & Maillard [23]. The DSB protocol consisted of three cycles of alternating growth under hydrated conditions for 48 h, followed by a further 48 h under dry conditions (Figure S1); all incubation periods were carried out at room temperature. Isolates were standardised to 1 × 10^6^ cells/mL in Roswell Park Memorial Institute (RPMI) media supplemented with 5% (v/v) foetal calf serum (FCS [Sigma‐Aldrich]) to simulate soiling and organic load. Growth under hydrated conditions was initiated by seeding cells in cell‐culture treated 24‐well polystyrene microtitre plates (Thermo Fisher Scientific). The subsequent dry phase consisted of the removal of all liquid from wells. Biofilms were washed once with sterile phosphate‐buffered saline (PBS [Oxoid]) to remove non‐adherent cells after the first hydration cycle. Characterisation of biofilms, including quantitation of viable cells and total biomass, and disinfection efficacy testing, was carried out after each dry phase, as described below. Where appropriate, biomass was harvested from plates by the addition of PBS and scraping the surface using a sterile pipette tip. All experiments were performed in biological triplicate on three independent occasions, unless otherwise stated.

### 2.3 Disinfectant Preparation

NaOCl disinfectant was purchased as a solution containing 5% or 50,000 ppm active chlorine and stored at 4°C until use. Working solutions of NaOCl were prepared fresh for each experiment by dilution in RPMI (Sigma‐Aldrich) or sterile water to concentrations between 4 and 4000 ppm.

### 2.4 Disinfectant Susceptibility Testing

Planktonic minimum inhibitory concentrations (PMICs) were determined for NaOCl adapted from the CLSI‐M27 broth dilution method [24]. In brief, NaOCl was serially diluted two‐fold in wells of 96‐well round bottom microtitre plates between 7.8 and 4000 ppm in RPMI, at double the final concentration. Planktonic cells were added to wells at a final concentration of 1 × 10^4^ cells/mL in RPMI, and plates were incubated at 37°C for 24 h. Microbial growth was assessed visually and by measuring the optical density at 530 nm using a spectrophotometric plate reader. The MIC was determined as the concentration of NaOCl required to inhibit 50% or 90% of microbial growth relative to untreated controls.

### 2.5 Disinfection Efficacy Protocol

The efficacy of NaOCl disinfection against planktonic and biofilm cells was determined according to a previously published method [3]. In brief, planktonic cells were initially standardised to 2 × 10^8^ cells/mL in PBS + 5% FCS and aliquoted into 24‐well microtitre plates. Biofilms were also grown in 24‐well microtitre plates for one, two, or three cycles of the DSB protocol. NaOCl was diluted from stock to 500–2000 ppm in sterile water, and cells and biofilms were treated with a final concentration of 500 ppm or 1000 ppm for 1 or 5 min. Sterile water was used as a positive control (untreated). The active agent was neutralised with sterile sodium thiosulfate solution (Na_2_S_2_O_3_; 5% w/v in H_2_O [Sigma‐Aldrich]) added to all samples for 15 min. Planktonic cells were treated with an equal volume of 2× NaOCl to cells and neutralisation was carried out using two volumes Na_2_S_2_O_3_. In contrast, 1× NaOCl was added directly to washed biofilms, and neutralised with an equal volume of Na_2_S_2_O_3_. Surviving cells were resuspended in fresh PBS following neutralisation for quantification using viable cell counts as detailed below.

### 2.6 Viable Cell Counts

The Miles and Misra technique [3] was used to quantify viable cells during the formation of DSBs or following the disinfection of planktonic cells and DSBs. Cells and biomass were collected and serially diluted 10‐fold in sterile PBS and cultured onto SAB plates in triplicate across each dilution. Plates were incubated for 48 h at 30°C. The determination of colony forming units (CFU) per mL was performed using the average colony count of replicates.

### 2.7 Quantification of Total Biofilm Biomass

Total biomass was measured at the end of each growth cycle using the crystal violet assay [25]. Washed DSBs were stained with 0.5% (w/v) crystal violet for 15 min, after which excess was removed and biofilms washed twice. Plates were dried before eluting bound dye with absolute ethanol, of which 75 μL was transferred to a 96‐well microtitre plate, and the optical density measured at 570 nm in a plate reader.

### 2.8 Scanning Electron Microscopy

DSB ultrastructure was imaged using scanning electron microscopy (SEM) [26]. Briefly, DSBs grown for one (Day 5) or three cycles (Day 12) on Nunc Thermanox coverslips (Fisher Scientific) were fixed using 2% paraformaldehyde, 2% glutaraldehyde, 0.15% alician blue powder and 0.15 M sodium cacodylate. Samples were counterstained using uranyl acetate with subsequent gradient dehydration in ethanol (30%–100%). Dried samples were mounted, sputter‐coated using gold/palladium and visualised using an IT 100 SEM machine at 1000× and 10,000× magnification (JEOL Ltd).

### 2.9 RNA Extraction and Sequencing

A total of 5 × 10^8^ cells/mL from isolates NCPF8973 and NCPF8978 were collected by centrifugation following overnight growth. DSBs were also grown for each isolate and biomass harvested by scraping and centrifugation as detailed in section dry surface biofilm formation. RNA was extracted from samples using the RiboPure Yeast kit (Invitrogen) according to the manufacturer's instructions. The yield and quality of RNA was assessed using a DS‐11 Fx + spectrophotometer (DeNovix, USA), and the RNA Integrity Number (RIN) determined by Bioanalyser (Agilent). All samples had a yield > 500 ng/μL and RIN ≥ 7.2, deemed acceptable for downstream applications. RNA sequencing (RNA‐seq) was performed by Novogene (https://www.novogene.com/) on a Novaseq 6000 platform (Illumina) to produce 150 bp paired‐end reads according to their standard protocols. The data presented in this publication have been deposited into NCBI's Gene Expression Omnibus (https://www.ncbi.nlm.nih.gov/geo/) and are accessible through GEO Series accession number GSE239851.

### 2.10 Transcriptomics Analysis

Transcriptional profiling using RNA‐seq data was carried out using R (v4.3.0) in RStudio (v32), and all packages used within are open‐source and available through the Comprehensive R Archive Network (https://cran.r‐project.org), Bioconductor (https://bioconductor.org), or GitHub (https://github.com). Data handling and graphical outputs were performed using tidyverse packages (v2.0.0). Genomic sequences, gene annotations and gene ontology (GO) annotations for *C. auris* calde I reference strain B8441 were accessed from the *Candida* Genome Database (v s01‐m01‐r31; http://www.candidagenome.org) and the GO Consortium (http://geneontology.org). Raw sequencing reads were quality controlled using FastQC (v0.12.1). Genome indexing and read mapping were carried out in Kallisto (v0.48.0). Abundance outputs were converted to log_2_‐transformed counts per million (logCPM), filtered and normalised to the library size using EdgeR (v3.42.4). Differentially expressed genes were selected based on log_2_‐fold change ≥ 1.5 (logFC) in either direction and a false discovery rate (FDR) significance *p* < 0.01, determined using limma‐voom (limma v3.56.1). GO overrepresentation analysis (hypergeometric distribution, FDR multiple comparisons *p* < 0.05) was performed on gene sets containing up and downregulated genes for each analysis using clusterprofiler (v4.8.1).

### 2.11 Data Analysis

Data were analysed in Microsoft Excel (v16.73) and graphs compiled in GraphPad PRISM (v9.1.0). Normality tests were performed prior to appropriate statistical analysis. Viable cells from disinfected biofilms were compared to untreated biofilms using one‐way ANOVA with the Benjamini, Krieger and Yekutieli two‐stage step‐up method for controlling FDR as post hoc analysis. Significance was set at *p* < 0.05.

## 3 Results

### 3.1 Susceptibility of Planktonic Cells to Sodium Hypochlorite

**FIGURE 1:**
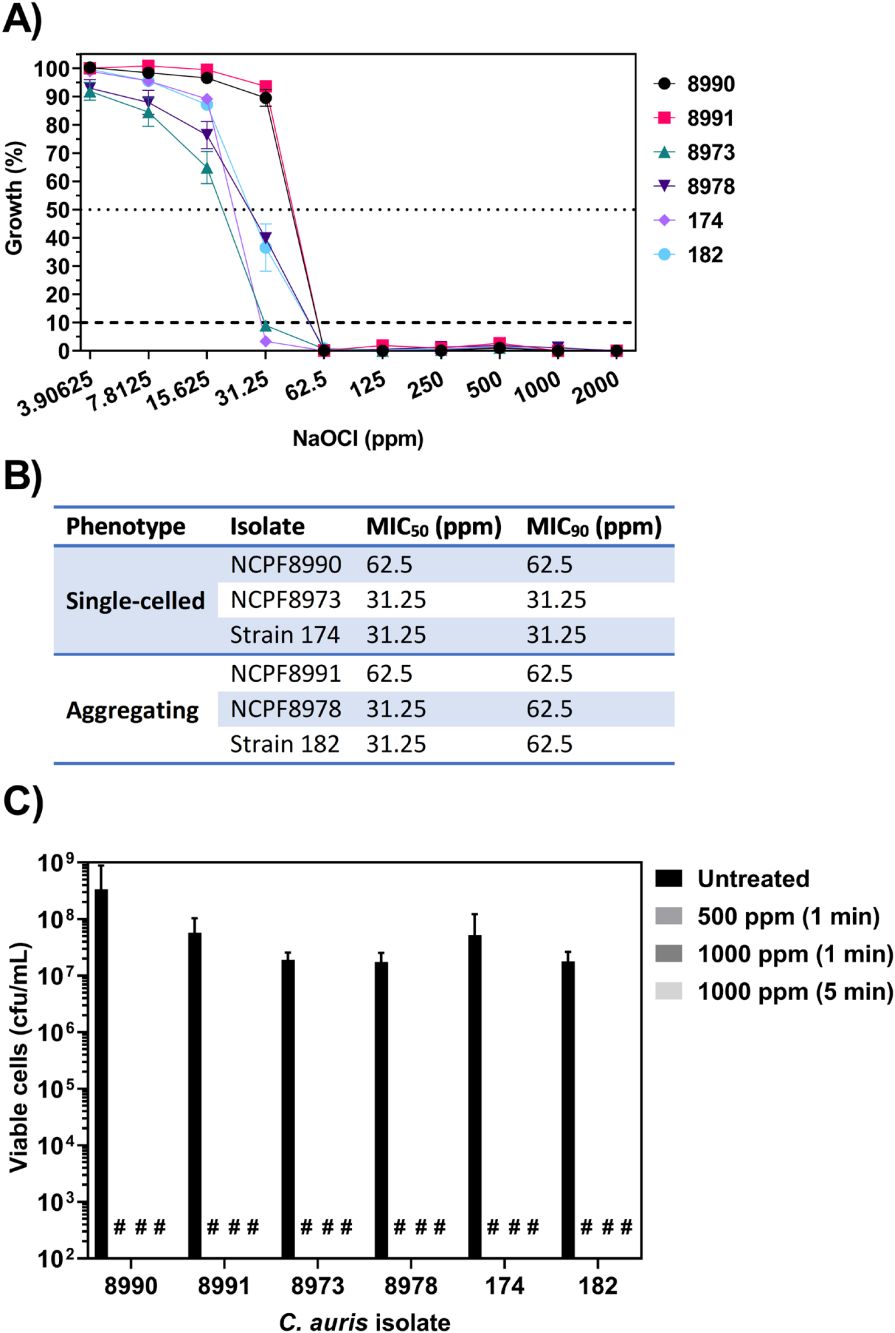
Susceptibility testing of *Candida auris* clinical strains against sodium hypochlorite. (A) Planktonic cells were co‐cultured with increasing concentrations of sodium hypochlorite (NaOCl) for 24 h. Growth of exposed cells was measured using absorbance at 530 nm and normalised to untreated cells for each strain. Dashed lines indicate growth below 50% and 10% of the positive control. (B) The minimum concentration of NaOCl required to inhibit 50% (MIC50) and 90% (MIC90) of cellular growth relative to untreated cells, was determined for each isolate. (C) Planktonic cells were treated with sodium hypochlorite (NaOCl) at 500–1000 ppm available chlorine for contact time of 1 or 5 min, or sterile water (untreated) for 5 min, before neutralisation of active agents. Data represent the mean (± SEM) for *n* = 3 experiments; # represents no cfu detected, limit of detection 50 cfu/ML.

Susceptibility to NaOCl has previously been reported for planktonic cultures of *C. auris* clinical isolates using a variety of methods [13, 15, 17, 27, 28, 29]. Intrinsic susceptibility to the chemical activity of NaOCl was confirmed in planktonic cells of six *C. auris* isolates of both aggregating and non‐aggregating phenotypes (Table 1). Activity of NaOCl following 24 h co‐culture was similar across the six isolates (Figure 1A). The MIC50 was shown to be 31.25 ppm for all isolates except NCPF8990 and NCPF8991, both of which had MIC50 of 62.5 ppm (Figure 1B). No growth was observed amongst isolates for concentrations of NaOCl above 62.5 ppm, which is well below the concentration recommended for routine cleaning [30].

The PMIC method for determining susceptibility does not accurately reflect all circumstances under which NaOCl is used as a disinfectant. We therefore evaluated the efficacy of NaOCl as a surface disinfectant when applied for 1 or 5 min, at manufacturer‐recommended concentrations of 500–1000 ppm active chlorine, and with neutralisation Na_2_S_2_O_3_ to standardise contact times. Treatment with NaOCl resulted in a ≥ 6‐log_10_ reduction in viable cells compared to the untreated cells across all six isolates, with no viable cells recovered after any treatment (Figure 1C).

### 3.2 Characterising Dry Surface Biofilm Formation by *C. auris* Isolates

**FIGURE 2:**
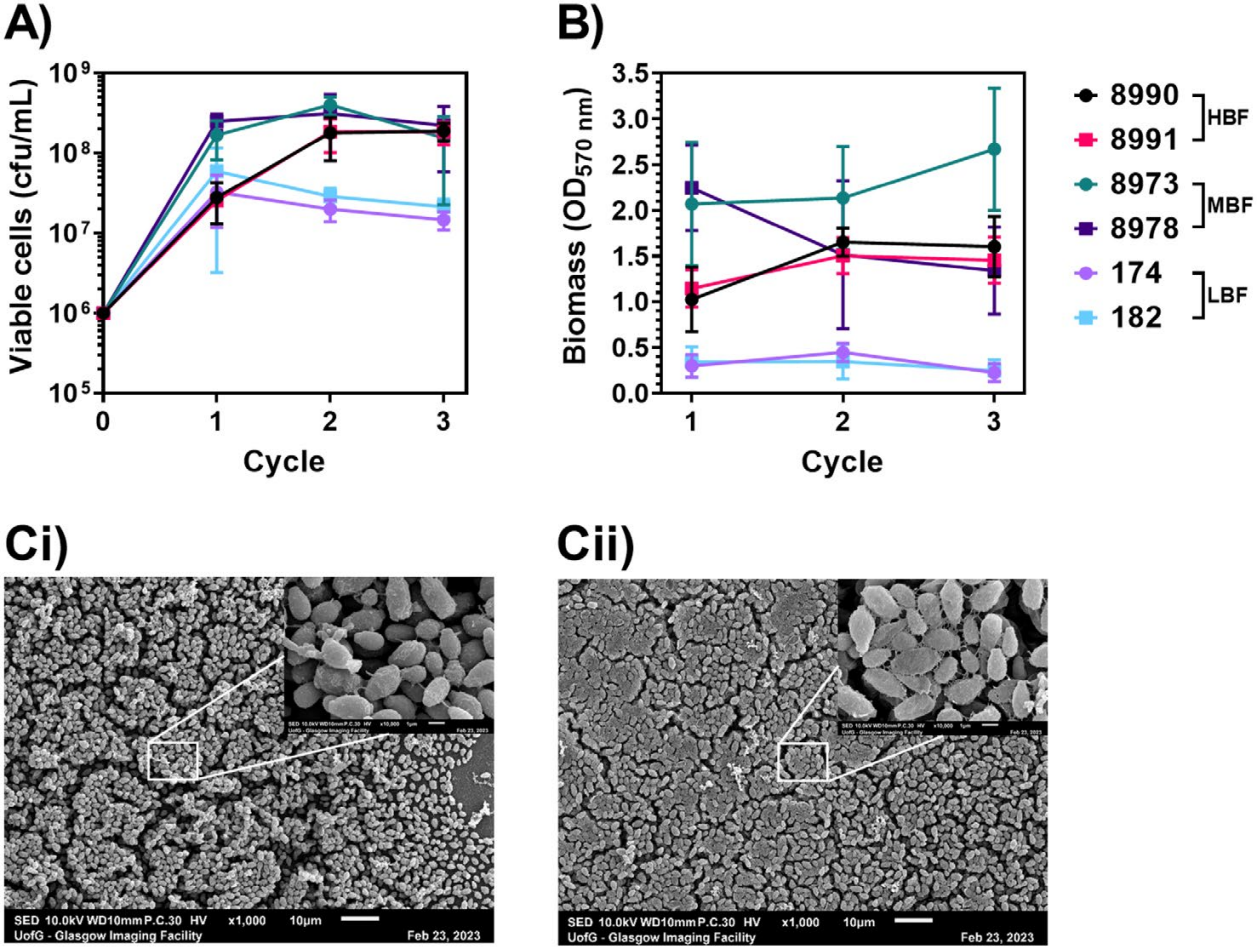
*Candida auris* form multilayer biofilms under dry surface conditions. (A, B) Clinical isolates of *C. auris* were standardised to 1 × 10^6^ cells/mL and grown as biofilms over one, two or three successive cycles of the DSB growth protocol. Viable cells (A) and biomass (B) were quantified at the conclusion of each cycle using plate counts and crystal violet assay, respectively. (C) Dry surface biofilms of single‐celled isolate NCPF8973 were grown on Thermanox coverslips over one (Ci) or three (Cii) successive cycles of wet/dry conditions. Biofilm ultrastructure was imaged using scanning electron microscopy at ×1000 and ×10000 (inset).

The DSB model has previously been used to simulate the growth of *C. auris* on stainless steel surfaces following contamination with biological material [23]. We screened the six different isolates for their ability to form biofilms on polystyrene surfaces under DSB conditions. Isolates were previously characterised as high‐, moderate‐, or low‐biofilm forming isolates using a 24 h model (HBF, MBF and LBF, respectively; Figure S2). All isolates developed biofilms using the DSB protocol, with viable cells and varying degrees of biomass detected in all strains across all time points (Figure 2A,B). The MBF isolates NCPF8973 and NCPF8978 demonstrated the greatest number of viable cells across all timepoints, which suggests they may be better suited to DSB formation than the other isolates. These two isolates were therefore selected for subsequent experiments. Using SEM, we observed dense, multi‐layered biofilms of isolate NCPF8973 across most areas of the substrate on Day 5 of growth, with other areas characterised by less dense monolayers of cells (Figure 2C). Higher magnification revealed the presence of ECM on the surface of some cells, with limited connections to other cells. By Day 12 of growth, the substrate was thickly covered with higher‐density, multi‐layered biofilms with interconnecting ECM covering the majority of cells (Figure 2C).

### 3.3 Development of NaOCl Tolerance in Dry Surface Biofilms

**FIGURE 3:**
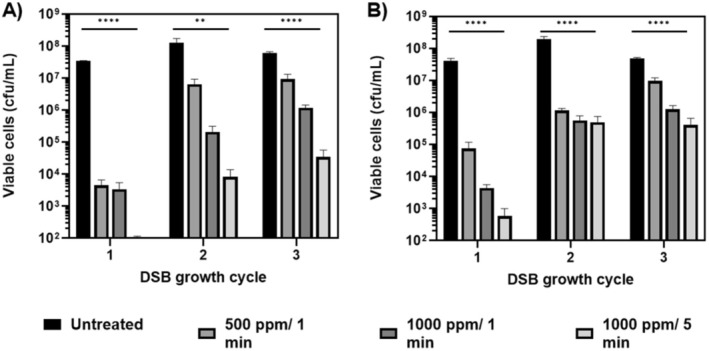
Repeated cycles of wet and dry conditions increase protection against sodium hypochlorite disinfection. *Candida auris* isolates NCPF8973 (A) and NCPF8978 (B) were grown as dry surface biofilms (DSBs) over one, two or three cycles of the protocol. At the conclusion of each cycle biofilms were treated with 500–1000 ppm sodium hypochlorite disinfectant for 1 or 5 min or sterile water (untreated) for 5 min. Data represents the mean (± SEM) viable counts for experiments performed in triplicate on three separate occasions. Treatments were compared to untreated biofilms using one‐way ANOVA with Benjamini, Krieger and Yekutieli two‐stage step‐up method for controlling false discovery rate post hoc analysis: **p < 0.01, ****p < 0.0001.

Given that *C. auris* biofilms display tolerance to antifungals and other small molecules [6], we further investigated the ability of *C. auris* NCPF8973 and NCPF8978 DSBs to withstand chemical disinfection with NaOCl. Across the three cycles, disinfection with NaOCl resulted in statistically significant reductions in viable cells for both isolates, regardless of the contact time and concentration of hypochlorite (*p* < 0.01; Figure 3). Each subsequent cycle, however, resulted in improved survival of disinfectant‐treated cells, evidenced by a decrease in log_10_‐reductions in viable cells for each treatment. Disinfection efficacy of 1 min at 500 ppm was most affected by subsequent growth of NCPF8973 DSBs, as the mean log_10_‐reduction in cfu/mL decreased from 4.5 to 1.2 from the first to the final cycle (Figure 3A). In contrast, the most affected treatment for NCPF8978 was 1000 ppm for 5 min, with a decrease in mean reduction in log_10_‐cfu/mL from 6.7 to 3.0 across the three cycles (Figure 3B).

### 3.4 Transcriptional Profiling of *C. auris* Dry Surface Biofilms

**FIGURE 4:**
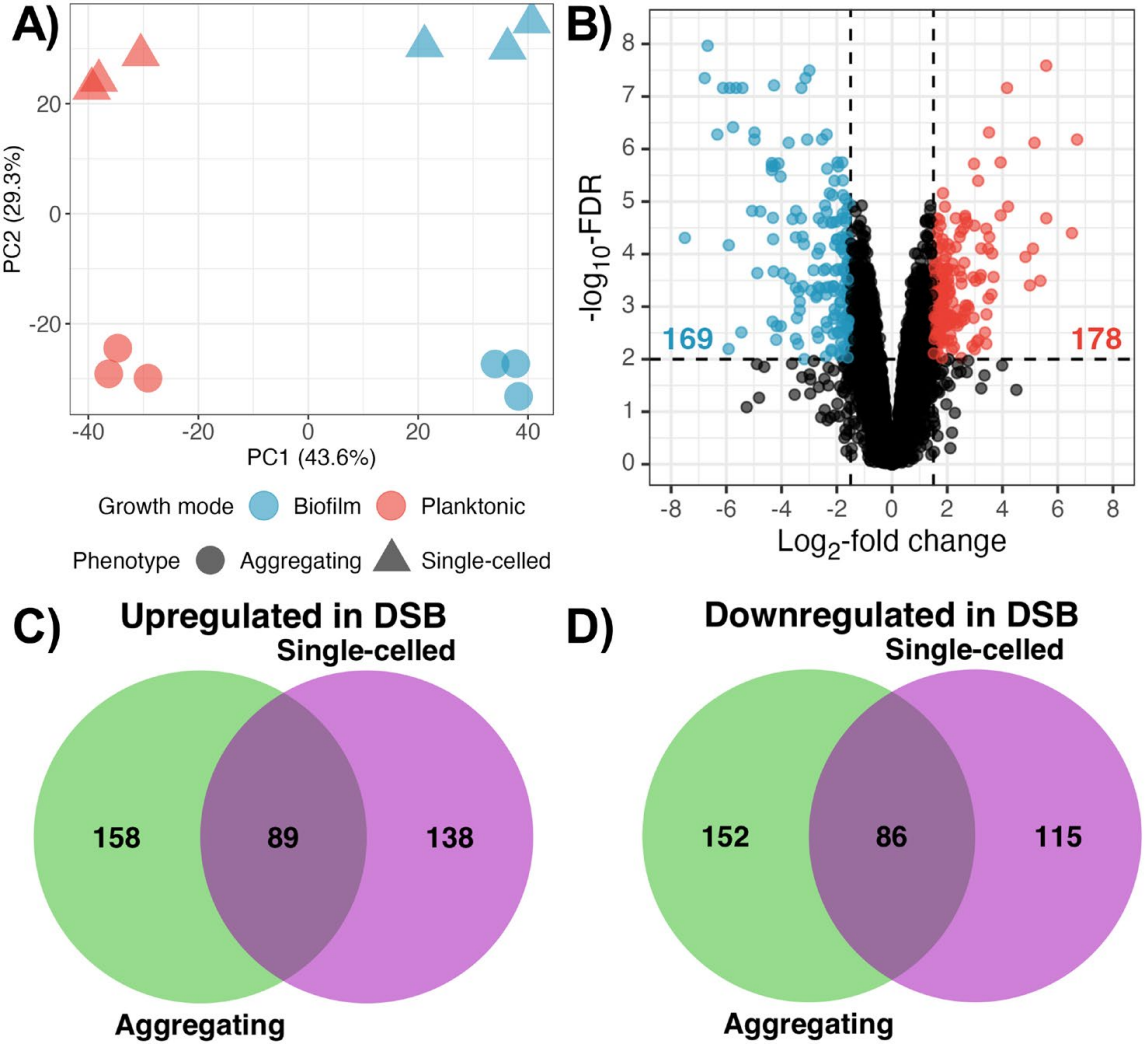
Gene expression by dry surface biofilms and planktonic cells is strain‐ and growth‐mode specific. RNA‐sequencing and transcriptional profiling was performed on cells grown for three cycles of the DSB protocol, and compared to planktonic cells. (A) Sources of variability between samples were examined by principal component analysis using filtered and normalised expression data. Principal Components 1 and 2, which accounted for over 70% of the variance, were plotted against each other, and samples coded by colour for growth mode, and shape for phenotype. (B—D) Differential gene expression analysis was carried out to determine up or downregulation of genes in DSB relative to planktonic cells, based on a log_2_‐fold change in expression of ≥ 1.5 with adjusted *p* values < 0.01. (B) Volcano plot of fold‐changes in gene expression between DSB and planktonic cells, versus probability of differential expression using combined data from both isolates; highlighted boxes show genes that are upregulated (blue) or downregulated (orange) in DSB cells relative to planktonic cells. (C and D) Venn diagrams of upregulated (C) and downregulated (D) genes between DSB and planktonic cells for single‐celled isolate NCPF 8973 and aggregating isolate NCPF8978.

RNA‐sequencing and transcriptomics analysis were performed on cells following planktonic and DSB growth to investigate mechanisms that could contribute to tolerance to disinfection. Total raw reads were > 10 million for each sample, average read alignment was > 90%, considered acceptable for transcriptomics analysis (Table S1). Principal component analysis (PCA) of expression data demonstrated that the greatest source of variance between samples (PC1) was the growth mode, namely DSB or planktonic conditions, followed closely by the isolate (PC2) as expected (Figure 4A). Differential expression (DE) analysis identified a total of 178 upregulated genes in DSBs relative to planktonic cells, and 169 downregulated genes (Figure 4B). Given that expression appeared to be isolate‐specific in the PCA, the number of DE genes between DSBs and planktonic cells was also examined for each individual isolate. In total, 227 and 201 genes were found to be up or downregulated, respectively, in the single‐celled isolate NPF8973 (Figure 4C,D), fewer than the 247 and 239 up or downregulated genes, respectively, in the aggregating isolate NCPF8978 (Figure 4C,D).

**FIGURE 5:**
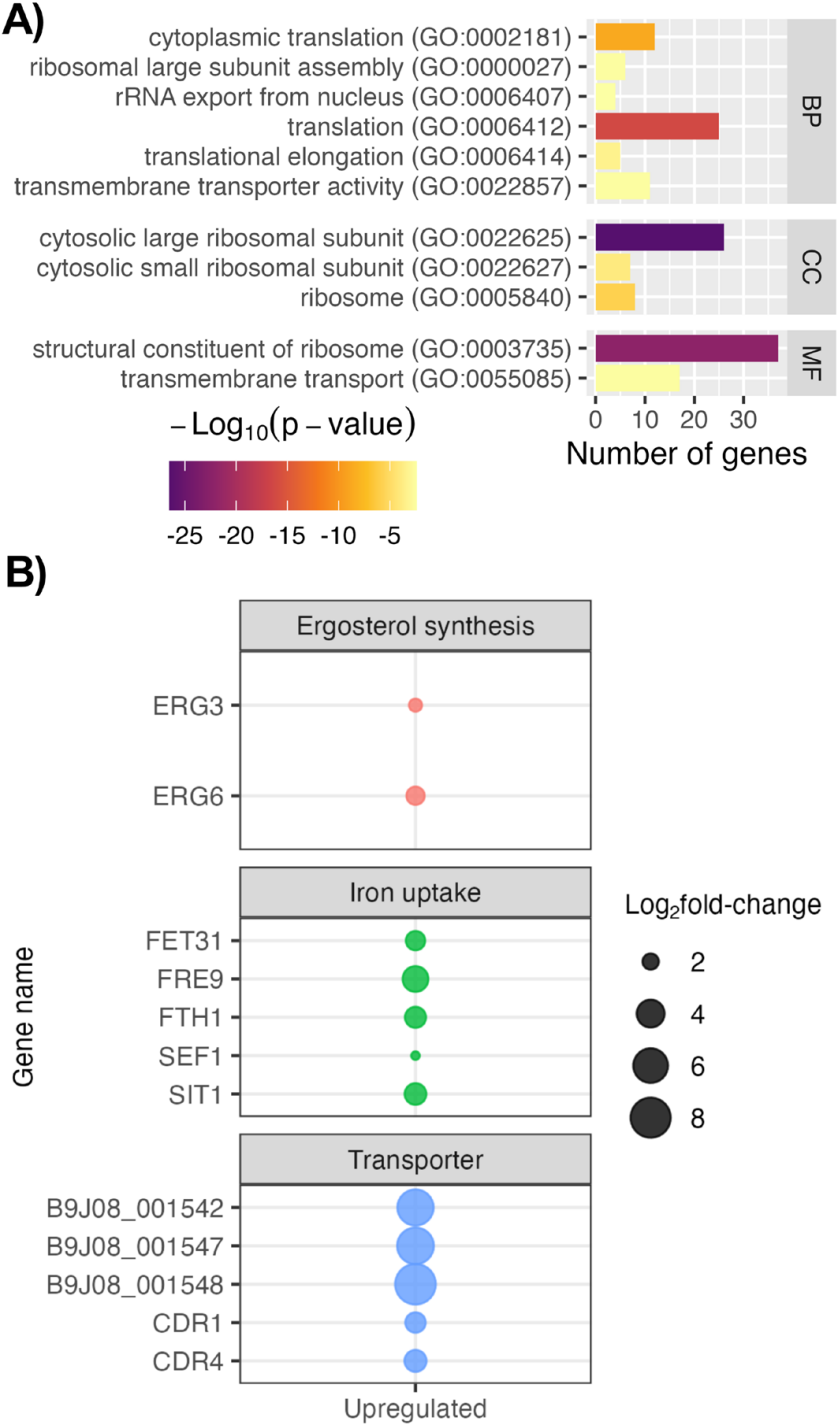
Dry surface biofilms upregulate expression of genes for transmembrane transport and iron uptake. (A) Gene ontology (GO) overrepresentation analysis was conducted on the 179 upregulated genes in DSBs and significantly enriched GO terms (*p* < 0.05, Benjamini‐Hochberg FDR multiple comparisons) were returned. (B) Genes of interest within the upregulated subset were further examined and assigned general categories based on their function. All analyses and figures were carried out in R. BP, biological process; CC, cellular component; MF, molecular function.

Gene ontology (GO) analysis identified 11 enriched terms amongst genes upregulated in DSBs for the combined‐isolate analysis (Figure 5A). Nine terms were concerned with ribosomal structure and assembly or translation, and the final two with transmembrane transport. Two ATP‐binding cassette (ABC) transporters, CDR1 and CDR4, as well as thirteen as‐yet uncharacterised open‐reading frames (ORFs), were identified amongst upregulated genes (Figure 5B), supporting the role of these genes in drug and, potentially, disinfectant tolerance. Cytoplasmic iron uptake was also upregulated by DSBs, including the siderophore transporter *SIT1*, ferric reductase *FRE9* and associated iron permease *FTH1*, ferroxidase *FET3/FET31*, and the transcriptional activator *SEF1* (Figure 5B). Seven uncharacterised ORFs thought to be members of the SIT1 family, which is expanded in *C. auris* relative to other Candida spp. [31], were also upregulated in DSBs (Table S2). This includes the two most highly upregulated ORFs B9J08_001548 (logFC = 8.2) and B9J08_001547 (logFC = 6.7), which remained in the top three most highly upregulated genes for each isolate, with similar log‐FCs (Table S2). Such strong and consistent upregulation of putative siderophore transporters highlights iron as a crucial factor in DSB formation by *C. auris* . Other ORFs, which are putative orthologues to ABC transporter *SNQ2* and major facilitator superfamily (MFS) transporters *BSC6* and *HGT2*, were also upregulated (Table S2). Genes for the synthesis of ergosterol (*ERG3* and *ERG6*; Figure 5B) and mannans (*MNN26*; Table S2), crucial for maintenance of the cell membrane and cell wall, respectively, were also amongst those upregulated by DSBs.

**FIGURE 6:**
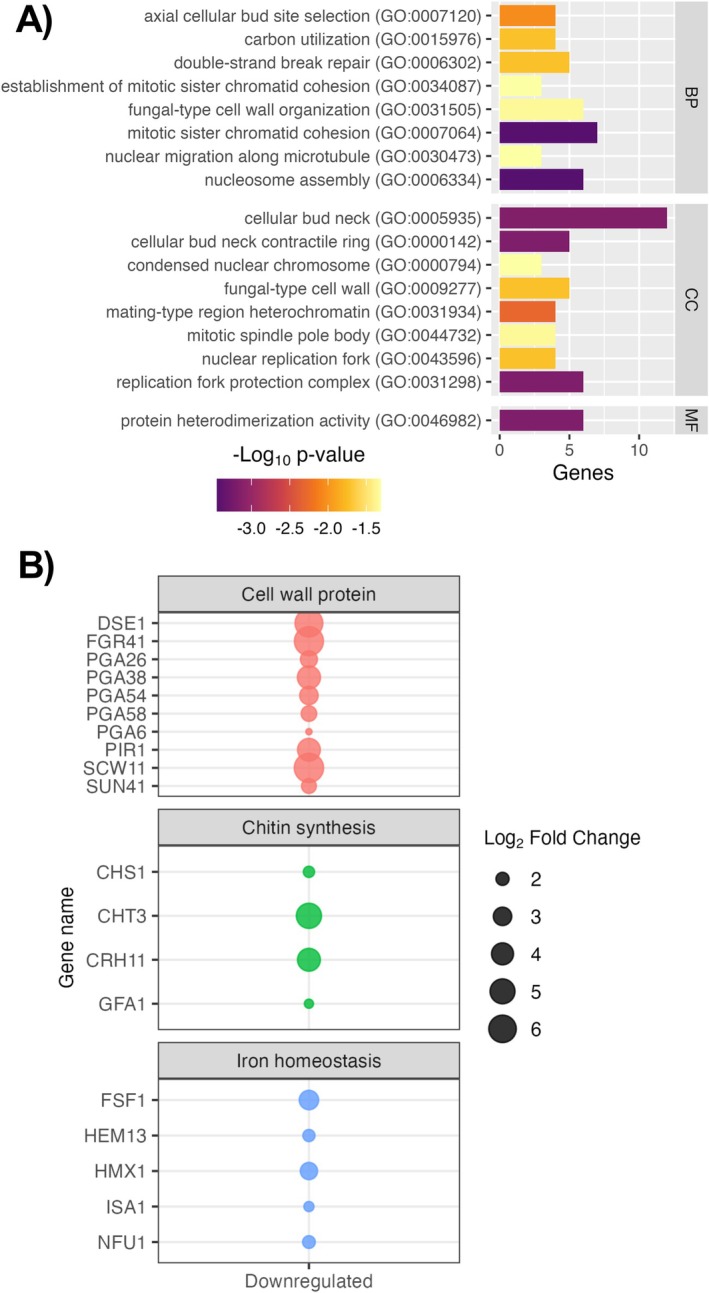
Expression of cell wall remodelling and haem utilisation genes are downregulated in dry surface biofilms. (A) Gene ontology (GO) overrepresentation analysis was conducted on downregulated genes within DSBs and significantly enriched GO terms returned (*p* < 0.05, Benjamini‐Hochberg FDR for multiple comparisons). (B) Genes of interest within the downregulated subset of genes were further examined and assigned categories based on their specific function. All analyses and figures were carried out in R. BP, biological process; CC, cellular component; MF, molecular function.

Overrepresentation analysis on genes downregulated by DSBs in both isolates found 17 enriched GO terms (Figure 6A), two of which were related to cell wall and its organization, and the remainder concerned with nuclear and/or cytoplasmic aspects of cell division. In contrast to iron scavenging pathways, a set of genes involved with mitochondrial iron homeostasis, haem biosynthesis and iron ion binding, *FSF1*, *HEM13*, *HXM1*, *ISA1*, *JLP1* and *NFU1* were found to be downregulated in DSBs (Figure 6B). Cell wall integrity proteins *PGA6*, *PGA26*, *PGA38*, *PGA54*, *PGA58*, *PIR1* and *DSE1* were all downregulated (Figure 6B). Cell wall remodelling enzymes *FGR41*, *SUN41* and *SCW11* for β‐glucans, *CHS1*, *CHT3*, *CRH11* and *GFA1* for chitin, and *ECM331* for mannan deposition were also downregulated (Figure 6B). *ACE2*, a transcription factor which activates *CHT3* and *SCW11* expression for cell separation, was also downregulated (Table S2). Downregulation of genes responsible for nuclear division and associated cell wall remodelling and separation also suggests that the DSBs have reached peak cellular density, consistent with the plateau in viable counts within DSBs following the first cycle of growth (Figure 2A). Collectively, the RNA‐seq data presented confirmed that DSBs contain metabolically active cells following the final cell cycle, although cellular division is less likely. Transmembrane transporter activity and iron homeostasis are potentially key processes in the formation and/or maintenance of dry‐surface biofilms in *C. auris* .

## 4 Discussion

The global emergence of *C. auris* since 2009 is unlike any other fungal disease [4], and poses a unique challenge within healthcare settings worldwide, where it continues to affect many vulnerable and at‐risk populations [9]. Many facilities where *C. auris* is endemic implement proactive screening approaches to detect colonisation and clinical disease, and prevent transmission [32]. Whilst such prioritisation has resulted in improved methods for detection of *C. auris* , many protocols for decontamination of colonised skin and abiotic surfaces are largely based on data from bacterial pathogens [30]. Failure to optimise disinfection protocols for *C. auris* could prolong outbreaks and exacerbate poor patient outcomes. As such, there is an urgent need for representative testing of disinfectants to guide development and implementation of cleaning and disinfection protocols within healthcare facilities.

In this study we have demonstrated that clinical isolates of *C. auris* are intrinsically susceptible to killing by NaOCl, using suspension testing of planktonic cells. Disinfection efficacy studies against planktonic cells typically demonstrate that *C. auris* isolates are susceptible to the chemical activity of chlorine‐based agents at concentrations ranging between 100 and 6500 ppm, depending on contact time [15, 16, 17, 27, 28, 29]. Contact times between 1 and 5 min achieve total eradication of viable cells using 100–1000 ppm chlorine‐based agents [15, 17, 29], consistent with our findings of ≥ 6‐log_10_ reductions with 500–1000 ppm NaOCl for the same time. When cultures are dried on surfaces, however, minimal reductions in log_10_‐cfu are observed with 500–2000 ppm NaOCl for 1 min [28], and concentrations of NaOCl ≥ 3900 ppm are required to achieve a minimum 4‐log_10_ reduction in cells with 1 min contact time [16, 27, 33]. Unlike studies on planktonic cells, varying degrees of susceptibility of *C. auris* biofilm communities to varying oxidizing agents have been reported. For example, early stage biofilms grown and treated with peracetic acid show substrate dependent susceptibility, with increased tolerance observed on stainless steel in comparison to cellulose and polystyrene [34]. Alternative oxidation‐based processes such as gaseous ozone and UVC have displayed varying effects between planktonic/vegetative cells and *C. auris* biofilms grown on polystyrene, with hybrid administration of both technologies needed to improve the overall decontamination efficacy [22]. Alongside antibiofilm activity, these automated methods have increased potential for reachability, can be eco‐friendlier, and can be used in combination delivery systems [21, 22, 35]. It is therefore evident that surface attachment and biofilm formation facilitate tolerance to disinfection of *C. auris* cells, further highlighting a need to optimize protocols taking into consideration both mode of growth and surface properties.

The seminal study previously characterising DSB formation by *C. auris* reported the development of sparse, monolayered biofilms lacking significant ECM [23]. These biofilms were effectively removed by mechanical disinfection with 1000 ppm NaOCl wipes, representing a ≥ 7‐log_10_ reduction in fungal burden. The clade II isolate used in their study, however, has since been shown to be more susceptible to NaOCl disinfection than other *C. auris* isolates [36], and clade II isolates are neither associated with outbreaks nor antifungal resistance [37]. In contrast, an isolate from an outbreak‐associated clade I used in our study formed thick, multilayer DSBs with extensive ECM over three cycles of growth. DSB development coincided with tolerance to the chemical activity of NaOCl, which was demonstrated by increased survival following successive cycles of growth. This tolerance occurred despite plateaued numbers of cells within DSBs after each cycle, further corroborated by downregulation of genes associated with nuclear replication and cell separation such as *ACE2* (B9J08_000468), SCW11 (B9J08_003120) and CHT3 (B9J08_002761). *ACE2* is a transcriptional activator of a number of genes including *SCW11*, *CHT3* and *DSE1*, which were all downregulated in DSBs. Interestingly, disruption of *ACE2* has been previously shown to induce cell separation defects resulting in aggregation [38]; in agreement with our data, this potentially suggests DSB formation occurs concurrent with aggregation induced by environmental stress and starvation [39].

The role of efflux pumps in establishing tolerance and/or resistance to antimicrobials has been widely documented in both bacterial and fungal species [40]. Multiple transporters belonging to *ABC* (Cdr1 and Snq2) and MFS (Mdr1 and Flu1) protein families are expressed by different *Candida* spp., and accept a wide variety of structurally diverse xenobiotics as substrates [40]. In vitro models of *C. auris* have demonstrated that constitutive expression of *CDR1* and *MDR1* by both planktonic cells and biofilms is strongly associated with increased MICs to azole antifungals [6, 41]. Furthermore, deletion of *CDR1* but not *MDR1* restored clinical susceptibility to fluconazole and itraconazole in resistant *C. auris* isolates, highlighting *CDR1* as a key player in tolerance to small‐molecule antifungals [41]. We also observed upregulation of *CDR1* (B9J08_000164) and *CDR4* (B9J08_000479), as well as ORFs with predicted transporter activity, by cells forming DSBs in our study, although a role for efflux pumps in NaOCl tolerance is unclear. Clinically relevant bacteria such as *Mycobacterium* spp. possess a redox‐sensing mechanism whereby oxidation of transcriptional repressors by intracellular NaOCl results in expression of efflux pumps, leading to subsequent removal of NaOCl molecules from cells [42]. Given that similar mechanisms have also been found in *Pseudomonas*, *Legionella* and *Bacillus* spp. [43], it is likely that *CDR1* and *CDR4* efflux pumps which are upregulated within *C. auris* DSBs facilitate removal of NaOCl and enable tolerance. Antifungal tolerance in *C. auris* biofilms also occurs due to the ECM components such as mannans, which have been previously demonstrated to actively sequester fluconazole molecules away from cells, thereby preventing its activity against cells [5]. In our model, *MNN26* (B9J08_004650) for mannan synthesis was upregulated, whilst *ECM331* (B9J08_004382) required for cell wall mannan deposition was downregulated, potentially suggesting that mannan synthesis was utilized extracellularly.

Iron homeostasis is critical to maintaining intracellular stores of the ions, which act as cofactors for many proteins and enzymes within cells [44]. Low iron conditions induce the expression of *SEF1* and *HAP43* transcriptional factors in *C. albicans* and *C. parapsilosis* , which in turn activate genes related to iron uptake and scavenging including *FET31*, *FTH1*, *FRE9* and *SIT1*, and turn off iron usage genes such as *HMX1*, and *HEM13* to maintain intracellular iron levels [44, 45]. Biofilm formation and virulence are also closely linked with iron homeostasis, as *SEF1* is required to maintain adhesion in *C. albicans* [46]. In our study, similar expression patterns were observed for the aforementioned genes, suggesting a conserved function of iron in maintaining *C. auris* biofilms.

Taken together, our data indicate that the *C. auris* isolates are capable of developing robust biofilms under the DSB protocol, and that extended growth under these conditions results in enhanced tolerance to NaOCl disinfection. Further functional studies into transmembrane efflux pumps such as CDR1, and iron homeostasis in DSBs are needed to elucidate mechanisms behind biofilm‐associated NaOCl tolerance which facilitate the persistence of this robust organism within the healthcare environment. Our work highlights the importance of selecting appropriate, clinically representative models to test the efficacy of disinfectants against nosocomial pathogens.

## 5 Conclusions

We have demonstrated that *C. auris* isolates from outbreak‐associated clades form robust dry surface biofilms in vitro. These biofilms exhibit increased tolerance to NaOCl disinfection over the course of development, resulting in loss of efficacy. Transcriptional profiling of these communities revealed that upregulation of small‐molecule efflux pumps and interference by the biofilm matrix are potential mechanisms by which *C. auris* DSB cells gain tolerance to NaOCl disinfection.

## Conflicts of Interest

The authors declare no conflicts of interest.

## Supporting information


**Figure S1.** The dry surface biofilm protocol. Isolates of *C. auris* were grown as dry surface biofilms (DSB) in appropriate growth medium over three cycles, each consisting of 48 h ‘wet’ phase where media was present, followed immediately by 48 h ‘dry’ phase wherein all media was removed from wells. Plates were incubated at room temperature at all times. Biofilms were assayed or treated at the conclusion of each dry phrase to determine the impact of each cycle on biofilm characteristics. Red lines indicate the beginning of each wet phase; brown lines indicate the beginning of each dry phase; blue dashed lines indicate the end of each cycle, at which time point characterisation assays were conducted (blue solid lines). Figure created using Biorender.com.

**Figure S2.** *Candida auris* clinical isolates show heterogeneity in biofilm forming ability. Twenty‐six clinical isolates were screened for biofilm formation. Cells standardised to 1 × 10^6^ cells/mL in RPMI were seeded into wells of 96‐well microtitre plates and biofilms grown over 24 h. Total biomass from washed biofilms was detected by staining with 0.5% (w/v) crystal violet solution and bound dye eluted in ethanol before quantification at 570 nm. Dotted and dashed lines represent cut‐off absorbance values for classification as high or moderate biofilm formers, respectively. Blue and green samples represent isolates which were selected for use in this study.

**Table S1.** Quality control of RNA‐sequence data from FASTQC and Kallisto.

Table S2.

## Data Availability

The data that support the findings of this study are openly available in https://www.ncbi.nlm.nih.gov/geo/ at https://www.ncbi.nlm.nih.gov/geo/query/acc.cgi?acc=GSE239851, reference number GSE239851.
