# Supplementary figures and images for: Dry Surface Biofilm Formation by Candida auris Facilitates Persistence and Tolerance to Sodium Hypochlorite
Source: APMIS. 2025 Apr 7;133(4):e70022. doi: 10.1111/apm.70022 (PMC11975465; doi:10.1111/apm.70022)

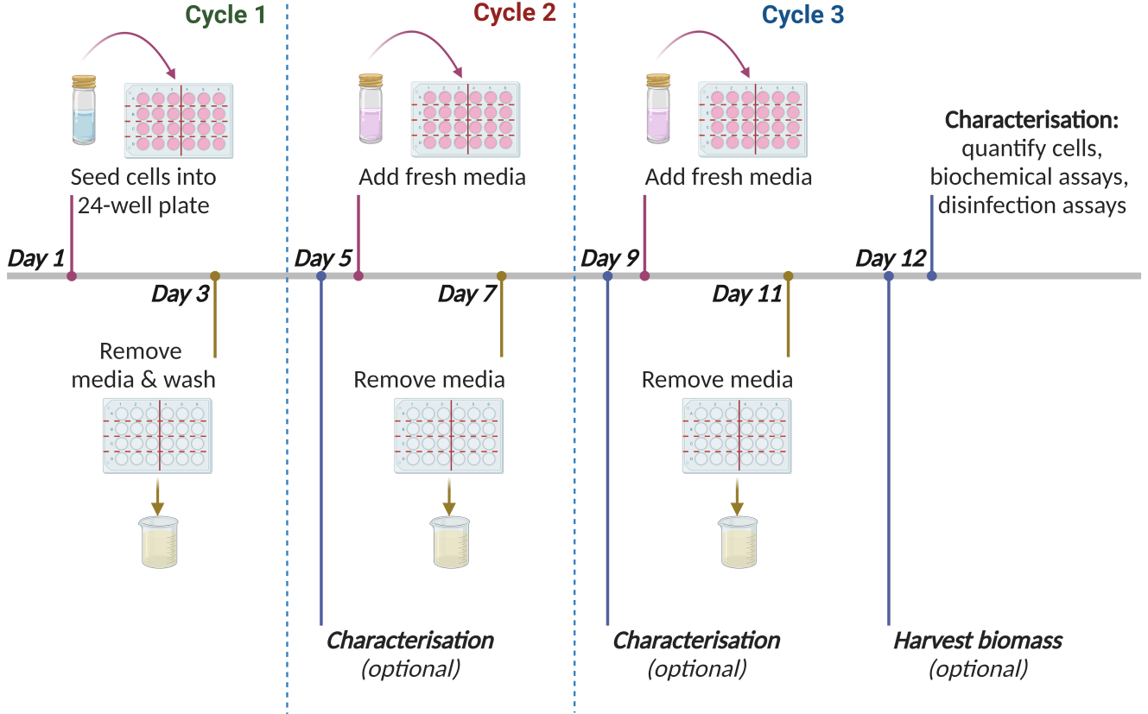

Supplement: Supplementary file 1 — Figure S1. The dry surface biofilm protocol. Isolates of C. auris were grown as dry surface biofilms (DSB) in appropriate growth medium over three cycles, each consisting of 48 h ‘wet’ phase where media was present, followed immediately by 48 h ‘dry’ phase wherein all media was removed from wells. Plates were incubated at room temperature at all times. Biofilms were assayed or treated at the conclusion of each dry phrase to determine the impact of each cycle on biofilm characteristics. Red lines indicate the beginning of each wet phase; brown lines indicate the beginning of each dry phase; blue dashed lines indicate the end of each cycle, at which time point characterisation assays were conducted (blue solid lines). Figure created using Biorender.com. [file APM-133-0-s002.tif]

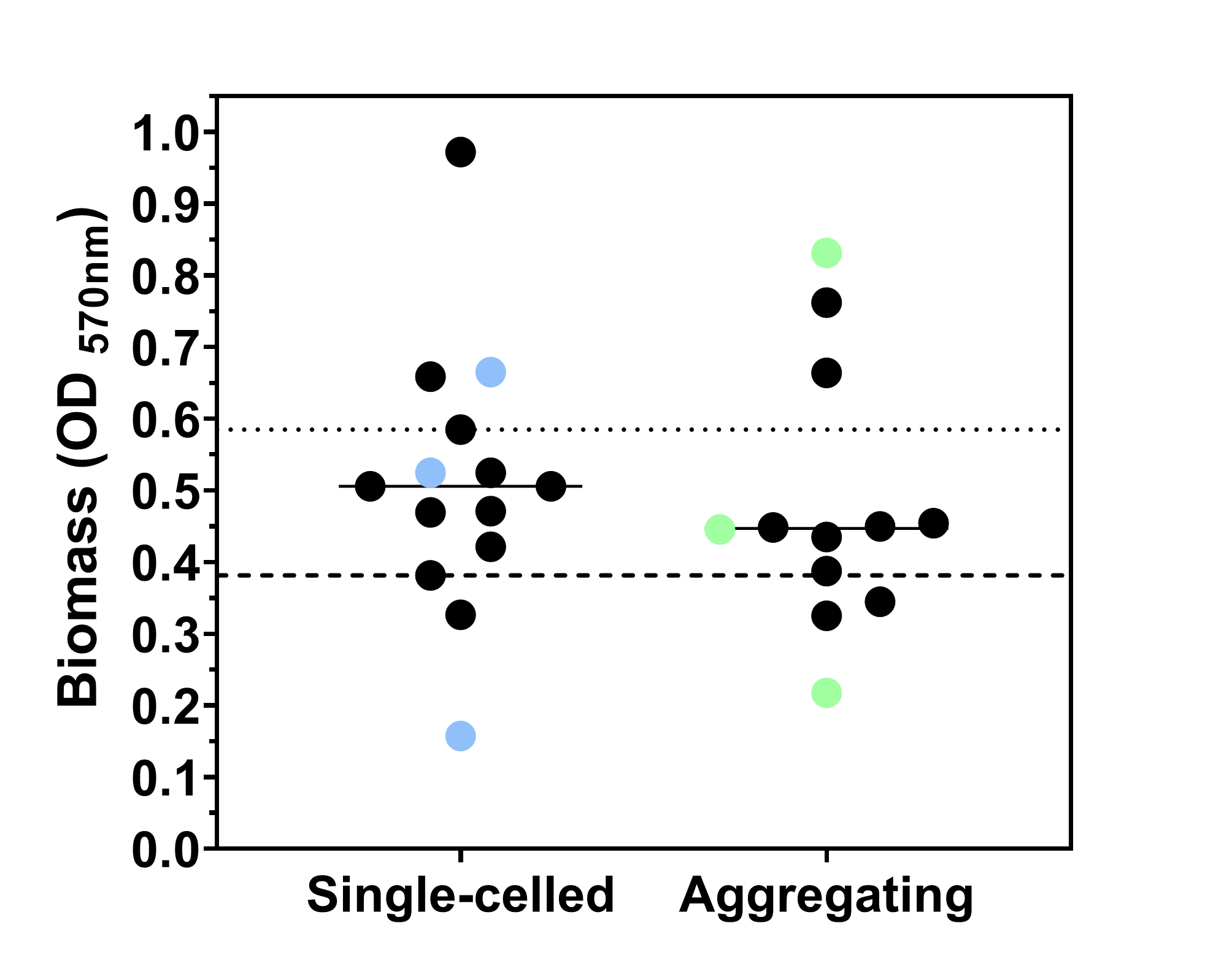

Supplement: Supplementary file 2 — Figure S2. Candida auris clinical isolates show heterogeneity in biofilm forming ability. Twenty‐six clinical isolates were screened for biofilm formation. Cells standardised to 1 × 10^6^ cells/mL in RPMI were seeded into wells of 96‐well microtitre plates and biofilms grown over 24 h. Total biomass from washed biofilms was detected by staining with 0.5% (w/v) crystal violet solution and bound dye eluted in ethanol before quantification at 570 nm. Dotted and dashed lines represent cut‐off absorbance values for classification as high or moderate biofilm formers, respectively. Blue and green samples represent isolates which were selected for use in this study. [file APM-133-0-s001.tif]
