# Supplementary material for: Dry Surface Biofilm Formation by Candida auris Facilitates Persistence and Tolerance to Sodium Hypochlorite
Source: APMIS. 2025 Apr 7;133(4):e70022. doi: 10.1111/apm.70022 (PMC11975465; doi:10.1111/apm.70022)
Supplement: Supplementary file 3 — Table S1. Quality control of RNA‐sequence data from FASTQC and Kallisto. [file APM-133-0-s003.docx]

**Table S1. Quality control of RNA-sequence data from FASTQC and Kallisto**

| **Sample** | **Bases (Gbp)** | **GC content (%)** | **FastQC warnings** | **Unique sequences (%)** | **Number of reads** | **Pseudoaligned reads** | **Read alignment (%)** |
| --- | --- | --- | --- | --- | --- | --- | --- |
| 73_PK_01 | 1.6 | 48 | *^1,2,3^* | 39.9-47.1 | 10738741 | 9462096 | 88.1 |
| 73_PK_02 | 1.5 | 48 | *^1,2,3^* | 38.4-44.2 | 10548340 | 9559205 | 90.6 |
| 73_PK_03 | 1.5 | 48 | *^1,2,3^* | 37.6-44.3 | 10521435 | 9507174 | 90.4 |
| 78_PK_01 | 1.9 | 48 | *^2,3^* | 37.8-43.0 | 12790891 | 11522627 | 90.1 |
| 78_PK_02 | 1.5 | 48 | *^1,2,3^* | 36.7-43.4 | 10585266 | 9624006 | 90.9 |
| 78_PK_03 | 1.5 | 48 | *^1,2,3^* | 36.4-43.4 | 10510911 | 9466833 | 90.1 |
| 73_SDB_01 | 1.9 | 48 | *^2,3^* | 35.3-41.1 | 13051803 | 11787166 | 90.3 |
| 73_SDB_02 | 1.8 | 48 | *^2,3^* | 37.4-43.2 | 12551926 | 11374048 | 90.6 |
| 73_SDB_03 | 1.5 | 48 | *^1,2,3^* | 37.9-44.5 | 10115912 | 9124434 | 90.2 |
| 78_SDB_01 | 1.7 | 48 | *^1,2,3^* | 36.6-44.9 | 11617808 | 10508297 | 90.4 |
| 78_SDB_02 | 1.8 | 48 | *^2,3^* | 38.5-44.1 | 12081946 | 10891720 | 90.1 |
| 78_SDB_03 | 1.8 | 48 | *^1,2,3^* | 38.6-46.1 | 12509477 | 11413115 | 91.2 |
| *^1^* Per tile sequence quality; *^2^* Per base sequence content; *^3^* Sequence duplication levels | | | | | | | |
